# Accuracy of a rapid diagnostic test on the diagnosis of malaria infection and of malaria - attributable fever during low and high transmission season in Burkina Faso

**DOI:** 10.1186/1475-2875-9-192

**Published:** 2010-07-07

**Authors:** Zeno Bisoffi, Sodiomon B Sirima, Joris Menten, Cristian Pattaro, Andrea Angheben, Federico Gobbi, Halidou Tinto, Claudia Lodesani, Bouma Neya, Maria Gobbo, Jef Van den Ende

**Affiliations:** 1 Centre for Tropical Diseases, S. Cuore Hospital, 37024 Negrar (Verona), Italy; 2 Projet AnKaHeresso, BP 292 Bobo Dioulasso, Burkina Faso; 3 Centre National de Recherche et de Formation sur le Paludisme, Ministry of Health, B.P. 2208, Ouagadougou 01, Burkina Faso; 4 Clinical Trials Unit, Prince Leopold Institute of Tropical Medicine, Nationalestraat 155, Antwerp, Belgium; 5 Institute of Genetic Medicine, European Academy (EURAC), Viale Druso 1 39100 Bozen/Bolzano, Italy; 6 Centre Muraz, BP 390, Bobo-Dioulasso 01, Burkina Faso; 7 Médecins Sans Frontières Italy, via Volturno 58, 00185 Rome, Italy; 8 Department of Clinical Sciences, Prince Leopold Institute of Tropical Medicine, Nationalestraat 155, Antwerp, Belgium

## Abstract

**Background:**

Malaria management policies currently recommend that the treatment should only be administered after laboratory confirmation. Where microscopy is not available, rapid diagnostic tests (RDTs) are the usual alternative. Conclusive evidence is still lacking on the safety of a test-based strategy for children. Moreover, no formal attempt has been made to estimate RDTs accuracy on malaria-attributable fever. This study aims at estimating the accuracy of a RDT for the diagnosis of both malaria infection and malaria - attributable fever, in a region of Burkina Faso with a typically seasonal malaria transmission pattern.

**Methods:**

Cross-sectional study. Subjects: all patients aged > 6 months consulting during the study periods. Gold standard for the diagnosis of malaria infection was microscopy. Gold standard for malaria-attributable fever was the number of fevers attributable to malaria, estimated by comparing parasite densities of febrile versus non-febrile subjects. Exclusion criteria: severe clinical condition needing urgent care.

**Results:**

In the dry season, 186/852 patients with fever (22%) and 213/1,382 patients without fever (15%) had a *Plasmodium falciparum *infection. In the rainy season, this proportion was 841/1,317 (64%) and 623/1,669 (37%), respectively. The attributable fraction of fever to malaria was 11% and 69%, respectively. The RDT was positive in 113/400 (28.3%) fever cases in the dry season, and in 443/650 (68.2%) in the rainy season. In the dry season, the RDT sensitivity and specificity for malaria infection were 86% and 90% respectively. In the rainy season they were 94% and 78% respectively. In the dry season, the RDT sensitivity and specificity for malaria-attributable fever were 94% and 75%, the positive predictive value (PPV) was 9% and the negative predictive value (NPV) was 99.8%. In the rainy season the test sensitivity for malaria-attributable fever was 97% and specificity was 55%. The PPV ranged from 38% for adults to 82% for infants, while the NPV ranged from 84% for infants to over 99% for adults.

**Conclusions:**

In the dry season the RDT has a low positive predictive value, but a very high negative predictive value for malaria-attributable fever. In the rainy season the negative test safely excludes malaria in adults but not in children.

## Background

The adoption of artemisinin-based combination therapy (ACT) for malaria in most endemic countries, and the availability of new diagnostic tools, such as rapid diagnostic tests (RDTs), have led the World Health Organization (WHO) to recommend a modified approach to malaria management. The previous policy indicated the presumptive treatment for malaria of all patients with fever, unless another obvious cause was found. The increased cost of new treatments, as well as the concern for the potential selection of drug resistant *Plasmodium falciparum *strains, prompted a more selective approach. The new policy recommends that malaria treatment should only be administered after laboratory confirmation. This recommendation had initially been limited to older children and adults, but the new edition of WHO guidelines, released on March 9 ^th^, 2010 [1], recommends to extend the test-based policy to children under five, too, as was strongly suggested by some authors [2]. There is no universal agreement on this new policy, though, and other authors argue that moving to a generalized test-based policy may not be safe, due to the sub-optimal performance of RDTs under field conditions, involving the risk of missing true malaria cases, with potentially fatal consequences in young children [3]. Moreover, recent field studies showed that the adherence of health workers to the test result was poor, with many patients being treated for malaria even after a negative test result, causing an obvious waste of resources [4-6].

Despite many published papers on RDT performance in different countries [7-20], important evidence gaps remain to be filled: a) the safety of a test-based strategy, especially for children, has not yet been fully demonstrated; b) evidence is needed on effective strategies to ensure an adequate compliance of prescribers with the test result; c) the utility of RDTs in areas of great seasonal variation in malaria transmission has not yet been adequately addressed, and the optimal policy might not be the same throughout the year [21]; d) although the accuracy of RDTs for malaria infection has been studied, no attempt has been made so far to estimate how accurately RDTs predict malaria - attributable fever.

A recent randomized trial on RDT-based versus presumptive treatment [4] aiming at assessing RDT safety on the same study population was partly frustrated, precisely, by a particularly poor adherence to the negative test result.

This paper addresses the third and fourth knowledge gaps, aiming at assessing RDT accuracy in an area of Burkina Faso with great seasonal variation in transmission intensity and at estimating, in both seasons, how RDTs perform as predictors of malaria-attributable fever. The two issues are clearly linked, as the proportion of fevers attributable to malaria may vary greatly according to the season.

RDTs are designed to detect malaria infection. In an endemic area many individuals are asymptomatic carriers of malaria parasites. A patient with fever and with malaria parasites in blood may be a case of malaria, or an individual with another cause of fever, and with incidental parasitaemia. No malaria test, including RDTs, is designed to discriminate between the two conditions, and indeed, any febrile patient with malaria parasites in blood, detected with whatever method, should be treated for malaria. However, if the parasite density is high, the fever is more likely to be due to malaria. Traditional microscopy provides a quantitative estimate of the parasite density, while RDTs do not, barely indicating the presence or absence of malaria parasites. A false negative RDT result may be of no consequence, if it fails to detect an incidental, low parasitaemia in a patient with another cause of fever. Paradoxically, a positive result might be harmful in the same patient, by confirming a clinical suspicion of malaria. RDTs based on HRP-2 protein have shown a variable accuracy for malaria infection in field studies, with microscopy taken as the gold standard. A recent review reported results ranging from 87.5% to 100% for sensitivity and from 52%% to 99.5% for specificity [15], the latter being also hampered by the tendency of the test to remain positive even weeks after a successfully treated malaria [10,22]. No study so far has formally attempted to assess RDTs as predictors of malaria-attributable fever.

## Research questions

This study primarily aimed at estimating how accurately RDTs predict malaria-attributable fever in the low and high transmission seasons. The main steps are outlined below:

1. The prevalence of malaria infection was assessed among febrile and non-febrile patients presenting at primary health care centres at the end of the dry and of the rainy season.

2. The fraction of fever episodes attributable to malaria infection (attributable fraction, AF) was determined in both seasons.

3. The accuracy of a rapid diagnostic test on malaria infection was evaluated in both seasons.

4. The performance of the RDT as a predictor of malaria-attributable fever was estimated in both seasons.

## Methods

A cross-sectional study was carried out in May and in October, 2006, in 10 primary care health centres of the provinces of Bobo Dioulasso and Banfora, south-west of Burkina Faso, an area with stable malaria and with a seasonal transmission pattern. The lowest transmission takes place at the end of the dry season (April-May), and the highest transmission at the end of the rainy season (October). The study sites were selected with convenience criteria, as described elsewhere [4].

All (febrile and non-febrile) patients > 6 months consulting one of the study sites for any clinical problem during the study periods (24^th ^April to 19^th ^May and 2^nd ^to 20^th ^October, 2006) and giving their (or their guardians') written informed consent were consecutively submitted to a standardized medical examination, and to thick and thin film, by specifically trained research assistants. The research assistants were trained by the study investigators and by professional laboratory staff from an Italian referral centre (see below) for three days preceding each study period: training included the correct execution of malaria smears (thick and thin film) and the execution and reading of the RDT.

Exclusion criteria were: severe clinical condition needing urgent care. An axillary temperature was obtained upon recruitment for all patients using an electronic digital thermometer (accuracy ± 0.1°C, certified CE 0197). Fever was defined as an axillary temperature ≥ 37.5°C.

A random sample of the febrile patients, in both seasons, was also submitted to a malaria RDT (Paracheck^® ^Device), in the context of the randomized trial cited above [4]. The febrile patients submitted to the RDT were, therefore, the same patients as in the trial, about half of the total febrile patients included in the study. The necessary sample size of the study was related to the primary outcome of the randomized trial: at least 2,000 febrile patients, plus all non-febrile patients presenting during the same periods.

The reference test was malaria microscopy executed by highly experienced staff from the Centre for Tropical Diseases (CTD) of S. Cuore Hospital of Negrar, Verona, a reference centre in Italy. The thick and thin films were coded locally and transported daily to a central laboratory (Centre Muraz, Bobo Dioulasso) for Giemsa staining by local staff, supervised by two senior microscopists from the CTD. Reading was done by the senior microscopists who were masked to the result of the RDT as well as to the clinical status (febrile or non-febrile) of the patients. A number of microscopic fields corresponding to 200 WBC were read in the thick film. The parasite density was calculated (for *P. falciparum *only) in the conventional way according with WHO criteria. A double blind cross reading of a random sample of 300 slides (thick plus thin films) was carried out in order to check for inter-observer variability, as a double reading of all the > 5,000 slides was not feasible.

The RDT used in the study was the test Paracheck^® ^Device (Orchid Biomedical Systems, Goa, India, batches 21,226 and 31,333, expiry dates June 2007 and January 2008, respectively), which detects the *P. falciparum *specific HRP-2 protein. The tests were individually sealed, were transported and stored according to the manufacturer's instructions, and were opened a few minutes before use. They were performed and read by the trained research assistants, coded and stored for future control. For positive results, the time of appearance of the positive band was recorded. RDT reading was checked every evening by the senior microscopists.

### Statistical analysis

Data were double-entered at Centre Muraz, Bobo Dioulasso, with Epi Info software (EpiInfo, CDC Atlanta, version 3.3.2). Data analyses were carried out with R 2.8.0 (R Development Core Team. R: A Language and Environment for Statistical Computing. R Foundation for Statistical Computing. Vienna, Austria, 2008. ISBN: 3-900051-07-0), and Stata 10.1 (StataCorp LP, College Station, TX 77845 USA) statistical packages.

The primary aim of the analysis was to estimate how RDTs predict malaria-attributable fever in the high and low transmission seasons. The main conceptual steps are summarized below, and detailed in the following paragraphs.

1. The prevalence of (falciparum) malaria infection was assessed in febrile and non-febrile patients.

2. The proportion of fevers attributable to malaria was estimated, stratifying by parasite density class and age group. The attributable fraction (AF) was defined as the proportion of fevers, among infected patients, that would not have occurred in the absence of malaria infection. Formulas used for AF calculation are reported below.

3. The diagnostic accuracy of RDTs for malaria infection by parasite density and age group was calculated.

4. The attributable fractions and the calculated diagnostic accuracy of RDT for malaria infection were combined to obtain an estimate of the sensitivity and specificity of RDT for malaria-attributable fever, accounting for both the expected increasing sensitivity of RDT for infection and the higher likelihood of a fever to be due to malaria at higher parasite densities.

All analyses were performed on the rainy and dry season data, separately.

The prevalence of falciparum malaria was estimated as the proportion of patients with a positive slide for *P. falciparum *asexual forms (any parasite density) among febrile and non-febrile patients. The AF of fever to malaria infection was estimated from the odds-ratios obtained from logistic regression modelling according to methods described for case-control studies [23] where patients with fever (axillary temperature ≥ 37.5°C) were defined as cases and patients without fever and without recent (3 days) fever history as controls. The odds-ratio (OR) of fever in each stratum of parasite density (0, 1-400, 401-4000, 4001-40,000 and > 40,000 parasites/μl, the upper limit of each stratum roughly corresponding to 1/10,000 parasites/RBC, 1/1,000, 1/100 and > 1/100) and age-group (6-11 months, 1-4 years, 5-14 years, ≥15 years) were calculated. The AF was then estimated from the ORs, as AF = (OR-1)/OR, for each cross-classification of parasite density and age (20 strata) for the rainy season. The number of fever cases and of positive malaria films was too low to determine the AF by age groups for the dry season. Consequently, for this season AFs were estimated by parasite density class only, adjusting for age, from the adjusted odds-ratios (aOR) as AF = (aOR-1)/aOR. In addition to the AF, the population attributable fraction (PAF) was also estimated, defined as the proportion of fevers attributable to malaria infection among all patients with fever, to assess the burden of disease in the whole population, and obtained by multiplying the AF by the prevalence of malaria infection among all febrile patients (PrevMal): PAF = AF(PrevMal).

RDT sensitivity, specificity, positive and negative predictive value (PPV, NPV) were estimated for malaria infection on the subset of febrile patients undergoing the RDT, and with microscopy results taken as the gold standard. As for the AF, sensitivity and specificity were calculated for each cross-classification of parasite density and age (20 strata) for the rainy season and for each parasite density stratum for the dry season. In addition, PPV and NPV of the RDT were assessed in febrile patients during the rainy and dry seasons. Confidence intervals were estimated with the Wilson's score method[24]. To assess how the agreement between RDT and microscopy was influenced by variables other than parasite density (such as season, age and sex), a logistic regression model was used where the outcome was a dichotomous variable taking values of 1 in case of method agreement or 0 in case of disagreement.

Based on RDT performances on malaria infection at each level of parasite density and age, its accuracy was subsequently assessed on malaria-attributable fever.

The AF-based approach does not allow classifying each individual febrile case with a positive slide as having clinical malaria, or simple malaria infection with another cause of fever. However, through this approach it is possible to estimate the number of malaria-attributable fevers at each stratum of parasite density and age, by multiplying the number of febrile cases in each stratum by the respective AF.

The number of true positive RDT results was then calculated in each stratum as the product of the number of malaria-attributable fevers and the probability of a RDT positive test result for febrile patients in the stratum. The total number of RDT true positives was the sum of the RDT true positives in all strata, as in formulas reported in Table 1a. In a similar way, the number of false positives (Table 1b), false negatives (Table 1c), and true negatives (Table 1d) were estimated. The RDT sensitivity was then calculated as the ratio of true positives (Table 1a) to the sum of true positives and false negatives (Table 1a + b), and the specificity as the ratio of true negatives (Table 1d) to the sum of true negatives and false positives (Table 1c + d). Similarly, the PPV was estimated as the number of true positives (Table 1a) divided by the number of RDT positives (Table 1a + c), and the NPV as the number of true negatives (Table 1d) over the number of RDT negatives (Table 1b + d).

**Table 1:** Formulas used for the estimation of the RDT sensitivity and specificity on malaria - attributable fever

	Malaria - attributable fevers	Fevers not attributable to malaria
RDT+	a) True Positives	c) False Positives
		
RDT-	b) False Negatives	d) True Negatives
		
		

All summations of the 20 combinations from the cross-classification of parasite density (5 strata: 0, 1-400, 401-4000, 4001-40,000 and > 40,000) and age (4 strata: 6-11 months, 1-4 years, 5-14 years, ≥ 15 years).

As a sensitivity analysis, the assessment of RDT diagnostic accuracy was repeated using logistic regression models for the risk of fever (to calculate AF) and for the probability of testing positive at RDT for each individual patient, including malaria infection status (yes/no), log-parasite density, and linear and quadratic terms for age. For the rainy season analyses, age/infection status and age/parasite density interaction terms were also included. The product of the AF and probability for an RDT positive result was added up for all febrile patients to obtain the diagnostic accuracy of RDT for malaria-attributable fever, similar to the stratified analysis.

The study protocol was approved by the "Comité National d'Ethique" (National Ethical Committee) of Burkina Faso (N. 2006-011 of 7^th ^April 2006). Written informed consent was obtained through the use of an information sheet with detailed explanation of the purpose of the study and the procedures involved. Once the clinical officer had decided that a patient was eligible for inclusion, a research assistant gave the explanation in local language, in the presence of at least one independent witness. In case of agreement, the informed consent form was signed both by the patient (or one of the parents in case of minors) and by the witness. For illiterate people the signature was replaced by the fingerprint.

## Results

**Figure 1:**
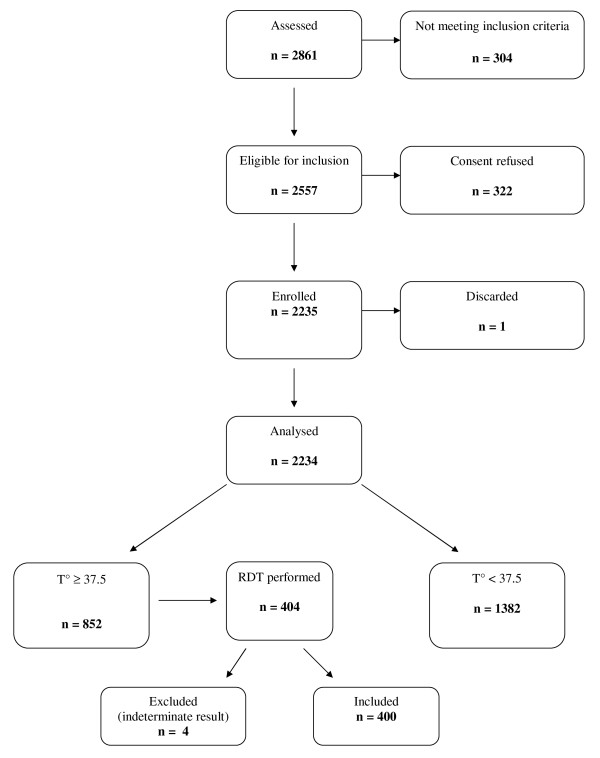
**Study flow chart, dry season**.

**Figure 2:**
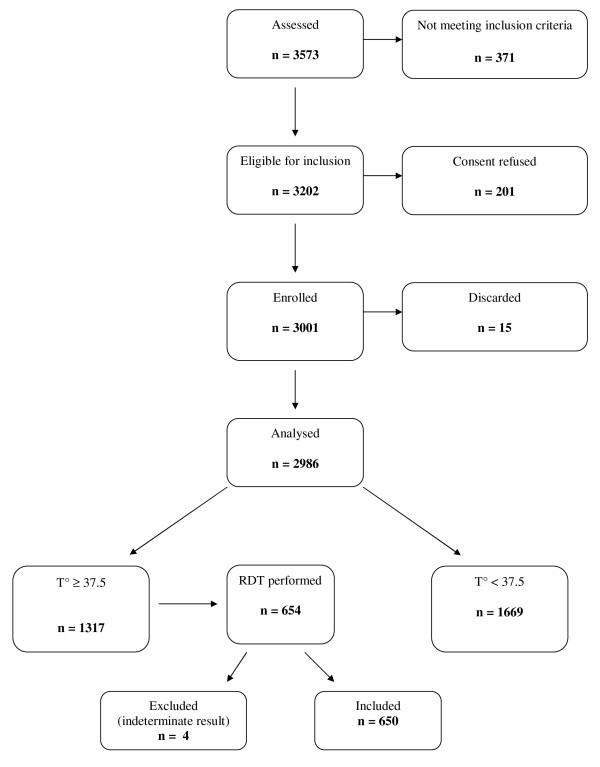
**Study flow chart, rainy season**.

A total of 5,759 patients eligible for inclusion (2,557 and 3,202 in the dry and rainy season, respectively) were asked their informed consent; 5,236 consenting patients were enrolled in the study (2,235 in the dry season and 3,001 in the rainy season) (Figure 1 and 2). Sixteen records (0.3%) were then excluded from the analysis because of missing data. The non-febrile patients were further classified according to fever history in the past three days: 474 and 731 patients with fever history and 908 and 938 without fever history in the dry and rainy season, respectively (Table 2).

**Table 2:** Characteristics of febrile and non-febrile patients included in the study

	Fever	**History of fever**^‡^	No fever
Dry season: N (%)	852	474	908
- Age: mean (SD)	11.5 (14.1)	22.2 (18.6)	22.7 (18.4)
- *P. falciparum *infected: n (%)	186 (21.8)	65 (13.7)	148 (16.3)
- median (IQR) parasite density^†^	360 (120 - 2520)	360 (120 - 2760)	240 (80 - 1300)
- RDT positive: n/N tested (%)	113/400^¥ ^(28.3)	NA	NA
Rainy season: N	1317	731	938
- Age (mean)	10.7 (13.4)	19.2 (17.4)	21.2 (16.8)
- *P. falciparum *infected: n (%)	841 (63.9)	305 (41.7)	318 (33.9)
- median (IQR) parasite density^†^	5200 (1124 - 42400)	2720 (360 - 15730)	600 (80 - 3320)
- RDT positive: n/N tests (%)	443/650^¥ ^(68.2)	NA	NA

^† ^For those with any parasites.

^‡ ^For those with axillary temperature < 37.5°C.

^¥ ^Seven indeterminate results (four in the dry season and three in the rainy season) and one missing result (rainy season) were excluded from the denominator of N tested and from subsequent calculations of RDT accuracy

### Microscopy and RDT reading

The inter-observer variability of microscopy reading was assessed on a randomly taken sample of 300 slides that were blindly re-read by the study microscopists. In one case, a slide previously read negative was subsequently read positive (presence of *P. falciparum *with parasite density < 50/μL, confirmed by a third reading), and conversely, in another case, a previously diagnosed very low *P. falciparum *parasitemia (< 50/μL) was missed by the second reading (the third reading confirmed the presence of *P. falciparum *asexual forms). Inter-observer variability in the assessment of parasite density was within acceptable limits: in particular, in three instances only was the class of parasite density (see below) different among observers, and the difference was of one class only. RDT reading by the research assistants presented no problem and was invariably confirmed by the study supervisors on site and by the subsequent control (every evening) by the senior microscopists. Only for seven samples (four in the dry season and three in the rainy season) the result was reported as indeterminate, and in one case (rainy season) it was missing. All eight were then excluded from the analysis of RDT accuracy.

### Prevalence of malaria infection and of positive RDT results

In the dry season, 186/852 patients with fever (22%), and 213/1,382 patients without fever (15%) had a *P. falciparum *malaria infection. Among the latter group, the proportion was 148/908 (16%) in the group reporting no fever history in the last three days, and 65/474 (14%) in the group reporting such history. In the rainy season, the proportion of patients with *P. falciparum *malaria infection was 841/1,317 (64%) and 623/1,669 (37%) in patients with and without fever, respectively; and in particular: 318/938 (34%) in the group with no fever history and 305/731 (42%) in the group with such history. The group with fever history was excluded from the calculation of the AF, in order to avoid misclassification of febrile/non-febrile patients. The RDT was positive in 113/400 febrile patients in the dry season (28%) and in 443/650 febrile patients in the rainy season (68%).

### Attributable fraction

The overall population attributable fraction (PAF) of fever to malaria among patients attending health clinics (obtained by multiplying the AF with the prevalence of malaria infection) was 2% in the dry season and 44% in the rainy season. Among those infected, the AF was 11% and 69%, respectively (Table 3 and 4).

**Table 3:** Attributable fractions (AF) and population attributable fractions (PAF) of fever to malaria by parasite density (dry season)

Parasite density	**Cases**^†^		**Controls**^**‡**^				
(/μL)	n	%^¥^	n	%^¥^	aOR	AF	PAF
0	666	78	760	84	1		
1-400	99	12	88	10	1.1	0.06	
401-4000	50	6	47	5	0.7	0£	
4001-40000	26	3	12	1	1.3	0.24	
40000+	11	1	1	0	7.0	0.86	
**Total**	852		908			0.11	0.02

^† ^Cases: with fever, axillary temperature ≥ 37.5°C at hospital visit.

^‡ ^Controls: without fever, axillary temperature < 37.5°C at hospital visit and no reported history of fever during the preceding 3 days.

^¥ ^Percentage of patients in a certain parasite density class among cases/controls. aOR: odds-ratio (of fever at each stratum of parasite density, versus uninfected patients), adjusted for age; AF: attributable fraction, calculated as: ;

^£ ^(AF by definition ≥ 0)

PrevMal = % febrile patients with malaria infection

PAF: population attributable fraction, calculated as: PAF = AF(PrevMal).

**Table 4:** Attributable fractions (AF) and population attributable fractions (PAF) of fever to malaria by parasite density and age group (rainy season)

Age	Parasite density	Cases^†^		Controls^‡^				
(years)	(/μL)	n	%^¥^	n	%^¥^	OR	AF	PAF
< 1	0	27	17	32	64	1		
	1-400	14	9	1	2	16.6	0.94	
	401-4000	40	25	9	18	5.3	0.81	
	4001-40000	41	26	4	8	12.1	0.92	
	40000+	37	23	4	8	11.0	0.91	
	**Total**	159		50			0.88	0.73
1 - 4	0	111	22	88	55	1		
	1-400	40	8	14	9	2.3	0.56	
	401-4000	132	26	32	20	3.3	0.69	
	4001-40000	100	20	21	13	3.8	0.74	
	40000+	121	24	6	4	16.0	0.94	
	**Total**	504		161			0.77	0.60
5 - 14	0	77	28	66	48	1		
	1-400	34	12	31	23	0.9	0^£^	
	401-4000	57	21	26	19	1.9	0.47	
	4001-40000	52	19	12	9	3.7	0.73	
	40000+	55	20	2	1	23.6	0.96	
	**Total**	275		137			0.59	0.43
15+	0	261	69	434	74	1		
	1-400	36	10	98	17	0.6	0^£^	
	401-4000	36	10	36	6	1.7	0.40	
	4001-40000	41	11	21	4	3.2	0.69	
	40000+	5	1	1	0	8.3	0.88	
	**Total**	379		590			0.40	0.12
All	0	476	36	620	66	NA^¶^		
	1-400	124	9	144	15	NA^¶^	0.29	
	401-4000	265	20	103	11	NA^¶^	0.62	
	4001-40000	234	18	58	6	NA^¶^	0.76	
	40000+	218	17	13	1	NA^¶^	0.94	
	**Total**	1317		938			0.69	0.44

^† ^Cases: with fever, axillary temperature ≥ 37.5°C at hospital visit.

^‡ ^Controls: without fever, axillary temperature < 37.5°C at hospital visit and no reported history of fever during the preceding 3 days.

^¥ ^Percentage of patients in a certain parasite density class among cases/controls.

OR: odds-ratio (of fever at each stratum of parasite density and age, versus uninfected patients); AF: attributable fraction, calculated as: ;

^¶ ^ORs not calculated as overall AF calculated from results by age-category.

^£ ^AF by definition ≥ 0.

PAF: population attributable fraction (calculated as showed in Table 3).

NA: Not applicable.

**Figure 3:**
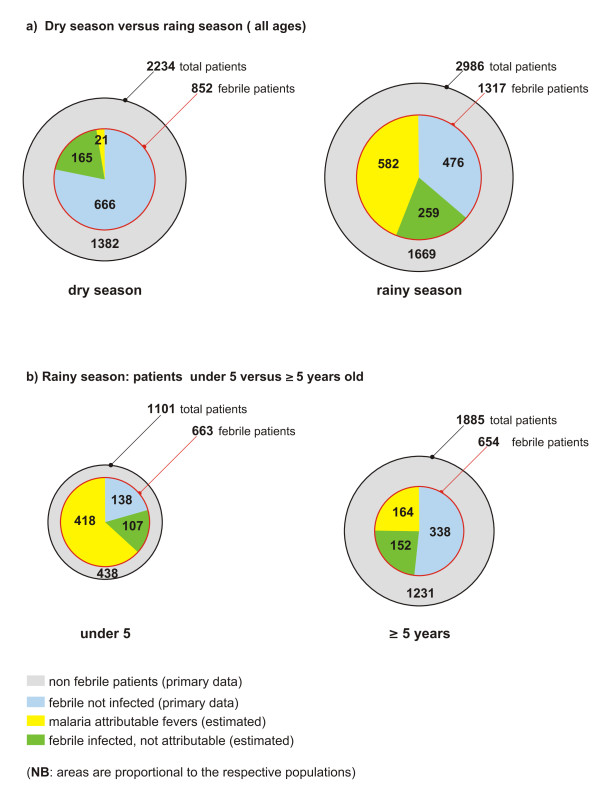
**Graphic representation of the patient population**.

In the dry season (Table 3), only in patients with over 4,000 parasites/μl was a considerable proportion of fever cases attributable to malaria (AF = 24% for parasite density between 4,001 and 40,000, and 86% for parasite density > 40,000). Results for the rainy season are reported in Table 4. AF was 29% for the lowest stratum of parasite density, 62% for density 401 to 4,000, 76% for density 4,000 to 40,000, and 94% for densities of > 40,000 parasites/μl. A visual breakdown of the whole patient population in the two seasons is reported in Figure 3a.

When results of the rainy season were stratified by age group, it was noted that AF decreased as age increased. For children younger than five years of age a substantial proportion of fever cases was attributable to malaria even at the lowest parasite density. In infants < 1 year old, in particular, even at the lowest parasite density (1-400/μl), the AF was very high (94%), and consistently so at all levels of parasite density. Contrarily to all other age groups, there was no substantial difference in AF across strata of parasite density (Table 4).

A visual breakdown of under 5 versus ≥ 5 years patient populations in the rainy season is reported in Figure 3b.

### Accuracy of RDT for malaria infection

The RDT was positive in 113/400 (28.3%) fever cases in the dry season, and in 443/650 (68.2%) in the rainy season (Table 2). Seven indeterminate results (four in the dry season and three in the rainy season) and one missing result (rainy season) were excluded from the denominator for calculations of RDT accuracy. Results from the logistic regression model showed, as expected, that the probability of agreement between RDT and microscopy was mostly influenced by parasite density. Season and age had little effect, while sex had no influence.

The sensitivity and specificity results of RDT for malaria infection are presented stratified by season, parasite density and age group. In the dry season (Table 5), overall sensitivity and specificity (when microscopy is taken as the gold standard) were 86% (95% CI: 78-92%) and 90% (95% CI: 86-92) respectively. In the rainy season, overall sensitivity was 94% (95% CI: 92-96) and specificity was 78% (95% CI: 72-83%) (Table 6). In both seasons, for parasite density below 400/μl test sensitivity was 76%, for densities between 400 and 4000 96% and 94% in the dry and rainy season respectively, and > 99% for higher densities. In one case, in the rainy season, a high parasite density (> 150,000/μl) was missed by the RDT. The patient, a six-year-old boy, was diagnosed as a case of malaria (the only symptoms were high fever and vomiting), but after the RDT result he was not given any antimalarial, but an antibiotic.

**Table 5:** Diagnostic accuracy of RDT for malaria infection by parasite density (dry season)

Parasite density		RDT Result			
(/μL)	N	positive (n)	negative (n)	Sensitivity (95% CI)	Specificity (95% CI)
0	306	32	274		90
1-400	50	38	12	76	
401-4000	28	27	1	96	
4001-40000	11	11	0	100	
40000+	5	5	0	100	

Not infected	306	32	274		90 (86 - 92)
Infected	94	81	13	86 (78 - 92)	
Total		113 (28%)	287 (72%)		

PPV during dry season: 72% (95% CI: 63 - 79%), NPV during dry season: 95% (95% CI: 92 - 97%).

**Table 6:** Diagnostic accuracy of RDT for malaria infection by parasite density and age group (rainy season)

Age	Parasite density		RDT Result			
(years)	(/μL)	N	positive (n)	negative (n)	Sensitivity (95% CI)	Specificity (95% CI)
< 1	0	15	6	9		60
	1-400	9	8	1	89	
	401-4000	18	17	1	94	
	4001-40000	22	22	0	100	
	40000+	20	20	0	100	
	Total	84 fdf(87%)(87%)	73 (87%)	11 (13%)		
1 - 4	0	57	22	35		61
	1-400	18	15	3	83	
	401-4000	69	66	3	96	
	4001-40000	50	50	0	100	
	40000+	56	56	0	100	
	Total	250	209 (84%)	41 (16%)		
5 - 14	0	37	13	24		65
	1-400	17	14	3	82	
	401-4000	30	28	2	93	
	4001-40000	20	20	0	100	
	40000+	24	23	1	96	
	Total	128	98 (77%)	30 (23%)		
15+	0	125	10	115		92
	1-400	19	11	8	58	
	401-4000	20	18	2	90	
	4001-40000	23	23	0	100	
	40000+	1	1	0	100	
	Total	188	63 (34%)	15 (66%)		

All	0	234	51	183		78
	1-400	63	48	15	76	
	401-4000	137	129	8	94	
	4001-40000	115	115	0	100	
	40000+	101	100	1	99	
	Total	650	443 (68%)	207 (32%)		

All	Not infected	234	51	183		78 (72 - 83)
	Infected	416	392	24	94 (92- 96)	

PPV during rainy season: 88% (95% CI: 85 - 91%), NPV during rainy season: 88% (95% CI: 83 - 92%).

The positive predictive value (PPV) for malaria infection in patients with fever was 72% (95% CI: 63-79) in the dry season and 88% (95% CI: 85-91) in the rainy season; the negative predictive value (NPV) was 95% (95% CI: 92-97) and 88% (95% CI: 83-92) in the dry and rainy season, respectively.

### Accuracy of RDT for malaria - attributable fever

In the dry season, RDT sensitivity and specificity for malaria-attributable fever were 94% and 75%, PPV was 9%, and NPV was 99.8%. In the rainy season, the test sensitivity for malaria attributable fever was 97% and was consistent across age groups, while specificity ranged between 36% (in children aged 5-14 yrs) and 78% (in adults), and was 55% overall. The PPV ranged from 38% in adults to 82% in infants, while the NPV ranged from 84% in infants to > 99% in adults. The overall PPV and NPV in the rainy season were 63% and 96%, respectively (Table 7). The breakdown by age group and parasite density is reported in Additional files 1 and 2. Results were confirmed by the sensitivity analysis with adjusted logistic regression modelling, as shown in Additional files 3 and 4.

**Table 7:** Estimated diagnostic accuracy of RDT for malaria - attributable fever during the low and high transmission seasons

Age		Febrile	Malaria-attributable		Not malaria-attributable		SE	SP	PPV	NPV
(years)		N	TP (a)	FN (b)	FP (c)	TN (d)				
*Low transmission (dry) season*										
< 1		143	4.4	0.2	35.9	102.4	95	74	11	99.8
1 - 4		299	8.8	0.5	88.2	201.5	94	70	9	99.7
5 - 14		130	4.0	0.3	39.9	85.7	93	68	9	99.7
15+		280	2.5	0.3	46.2	230.9	89	83	5	99.9

All		852	19.8	1.3	210.3	620.6	94	75	9	99.8

*High transmission (rainy) season*										
< 1		159	113.6	3.3	25.5	16.7	97	40	82	84
1 - 4		504	293.2	7.7	130.2	72.9	97	36	69	90
5 - 14		275	113.4	4.0	99.6	58.1	97	37	53	94
15+		379	45.7	1.4	74.4	257.4	97	78	38	99.4

All		1317	565.8	16.4	329.7	405.1	97	55	63	96

N: number of febrile patients in each age-parasite density combination.

TP, FN, FP, TN: expected number of true positives, false negatives, false positives and true negatives of the RDT for clinical malaria diagnosis among the N febrile cases in each age-parasite density combination. Estimates obtained from N, AF and Prob RDT + (see methods). Numbers presented are rounded to 1 decimal place; actual calculations based on a better numerical precision.

SE, SP, PPV, NPV: estimated sensitivity, specificity, positive predictive value, negative predictive value of RDT for clinical malaria.

## Discussion

According to microscopy, in the rainy season versus the dry season, the prevalence of malaria infection in patients presenting at primary health care centres was three times higher in febrile patients and twice in non-febrile patients. In the dry season, only a small proportion of fevers were attributable to malaria. This may be surprising, but is coherent with a low transmission level, typical of the dry season. In a previous study in Burkina Faso capital city Ouagadougou during the "cold" dry season, the AF of fever to malaria infection was also exceedingly low [25]. Conversely, in the rainy season, it was observed that almost half of all fevers were attributable to malaria and this proportion was highest in infants and lowest in adults. In infants (6-11 months) in the rainy season, our results clearly show that even at the lowest parasite density the attributable fraction is close to 100%: a fever associated with the presence of malaria parasites in blood is virtually always attributable to malaria in this age group, regardless the parasite density. A similar result was found by McGuinnes *et al *in Ghana [26]. In children 1 to 4 years old, only about half of the cases were attributable to malaria for densities < 400, while this proportion increased with parasite density. In older children and in adults, a fever was never attributable to malaria at parasite densities < 400. These findings are clearly relevant to the policy of RDT use to be adopted locally.

### RDT accuracy for malaria infection

The test sensitivity, specificity, positive and negative predictive values (PPV, NPV) for malaria infection, as reported in Tables 5 and 6, are within the range found by previous studies [15,20,27].

Although the different test sensitivity in the dry and in the rainy season may be surprising, this difference appears to be caused almost entirely by the different mean parasite density in the two seasons: if the analysis is stratified for parasite density, the sensitivity is very similar in both seasons. The overall sensitivity was lower than 95%, the minimal level recommended by the WHO. However, most false negative results occurred at the lowest parasite density. Over 400 parasites/μl the sensitivity was higher than 95% and approached 100% over 4,000 parasites/μl. Leaving without treatment patients with false negative results at low parasite density might be relatively harmless. Niama-Meya *et al *in Uganda showed that the missed treatment for patients with a false negative malaria microscopy never resulted in severe disease [28].

Unfortunately, even at very high parasite density the RDT sensitivity was not 100%. In one of our cases (a 6-year-old child in the rainy season), who had malaria with very high parasite density (> 150,000/μl), the RDT was negative and was confirmed as such by expert reading. This worrisome occurrence, though rare, has been described by others for HRP-based tests [29], and might be explained by the pro-zone effect [30,31]. It makes clear-cut policies for patient management even more problematic. In our study RDTs showed a disappointing specificity, particularly in the rainy season. It is well known that HPR-based tests such as Paracheck^® ^may remain positive for weeks after disappearance of trophozoites. A recent study showed that 70% of these tests were still positive 35 days after appropriate treatment [22]. Moreover, the gold standard, the thick film, at the reading standard of our study, had a detection limit of about 50 parasites/μl: some of the "false positive" RDT results may rather be false negative thick films. Studies with PCR have shown that RDT sensitivity for malaria infection may be higher than that of standard microscopy [32,33].

### RDT accuracy for malaria - attributable fever

In the dry season, after a positive RDT the probability for a fever to be attributable to malaria remains below 10%. It is unquestionable that all patients with a positive RDT should be treated for malaria. A positive RDT, however, should not influence the treatment decision for other, potential causes of fever. In settings/seasons where malaria accounts for a negligible proportion of all fevers, RDTs are claimed to be most useful, and safe [2,14]. This study confirms that a negative test brings the probability of malaria down to virtually zero in the dry season. As only 28% of all RDTs were positive, a correct use of the test would avoid an unnecessary treatment in three quarters of the fevers. However, the risk involved in false positive results has not been given the attention it deserves. As it was previously reported, mortality was significantly higher in the dry season, when no death was due to malaria, and in one of the fatal cases the RDT had given a (false) positive result [4].

The picture radically changes in the rainy season. Almost 90% of febrile children below 1 year, and almost 85% of those between 1 and 4 years, had a positive RDT (Table 6). After a positive test, a fever is very likely to be attributable to malaria (PPV 82% and 69%, respectively) (Table 7), and even after a negative test the disease cannot be ruled out in either group, with a residual probability of 16% and 10% (NPV 84% and 90%), respectively. This raises concern especially for infants (6-11 months), because, even at very low parasite densities (the most likely to go undetected by the RDTs), the fever is almost invariably attributable to malaria. On the contrary, the negative test virtually excludes malaria in older children and adults. As, in contrast to children, two third of adults have a negative RDT result (Table 6), there appears to be a strong rationale for recommending a test-based policy for this age group, which would save a substantial proportion of unnecessary malaria treatments.

No published study as yet has attempted to assess RDT performances on malarial fever rather than on simple malaria infection. A recent study in Mali used a different approach to estimate RDT accuracy for clinical malaria: the latter was defined with empirical criteria, using as a case definition either the diagnosis by a clinician, or the presence of fever and of ≥ 2000 plasmodia/μl at thick film [20]. Both case definitions are arguable, however. Malaria cannot be demonstrated on clinical grounds only [34,35]. The case definition based on parasite density is more adequate, but the optimal cut-off value should be assessed locally [36,37]. Any cut-off based method, however, leaves by definition a proportion of true malaria cases below the cut-off, and vice versa. The alternative method used in this study allows for an estimate of the actual number of malaria-attributable cases of fever within each stratum of parasite density.

### Strengths and weaknesses

This study provides for the first time a formal estimate of how RDTs predict malaria-attributable fever in a population exposed to a highly variable malaria transmission intensity across seasons. Assessing the RDT accuracy only on infection is of course methodologically easier, but less informative. If RDTs are able to exclude malarial fever when negative, then they can be considered relatively safe even when missing some infections; if they are positive in a high proportion of febrile cases that are not attributable to malaria, then it is crucial to train health care providers not to use the positive test result as a pretext for exclusion of other possible causes of fever.

Some limitations must be duly acknowledged. First, the choice of a study setting based on health facilities may be questioned as not being representative of the general population. The use of RDTs in the country is not planned at community level but in health facilities only, and therefore the study population is a sample of the population which is actually targeted for the test-based policy.

Alternative methods to the AF approach to the case definition of malarial fever have been suggested [38]. However, almost all papers published since 1991 [26,35-37,39-43] have used the AF calculation to this purpose. Of course, AF estimates can by no means be used as individual diagnostic criteria.

Errors (confidence intervals for sensitivity, specificity and predictive values of RDT) are presented in our manuscript only for malaria infection and not for malaria-attributable fever, due to a number of approximations for which the sampling error cannot be determined.

Finally, this study concerned but one out of the many commercially available RDTs that are currently under WHO scrutiny: several tests were more sensitive than Paracheck^® ^[22]. Would a more sensitive test change the main conclusions? The authors think it wouldn't. In the dry season, the test sensitivity on malaria-attributable fever was already optimal: a test with a better sensitivity, and equivalent specificity, for malaria infection would simply find more, clinically irrelevant, infections at low parasite density. In the rainy season, the same test might improve the safety of the test-based approach in young children, by identifying more cases of fever caused by malaria at the lower density strata, but at the expense of detecting also more cases of incidental parasitemia. As with Paracheck^® ^almost 90% of test results were positive, with a more sensitive test this percentage would be close to 100%, making it totally useless as a decisional tool.

### Policy implications

RDTs appear to be most useful during the low transmission season: a negative test safely excludes malaria and would avoid most unnecessary treatments, if prescribers are convinced to rely on the negative result. However, they must also be aware of the low predictive value of a positive test: clinical guidelines should not limit to indicate malaria treatment in this case, but clearly recommend considering other life-threatening diseases, regardless of the test result.

In the high transmission season a negative test does not safely exclude malaria in children below 5 years and particularly so in infants. Moreover, most tests are positive in febrile children. Although cost implications are beyond the scope of this paper, it is clear that in such context the cost of the tests would be simply added to that of treatment, with a waste of resources. If these findings were confirmed by other studies, a RDT-based policy should not be recommended in similar contexts for young children. Therefore, in areas with great seasonal variation in malaria prevalence, the optimal policy might not be the same throughout the year. While for adults the indications would not differ, the same is not true for children. From an operational point of view, however, it is unrealistic to suggest a differentiated policy according to the season.

### Future research

The cost-effectiveness of a RDT-based policy, in comparison with presumptive treatment, will be investigated, on the same study population, considering the test performances on malaria-attributable fever and not simply on infection.

## Conclusions

Despite the study limitations that are duly acknowledged, these data provide enough evidence to suggest extreme caution before moving to a generalized test-based policy in all contexts. In areas similar to this study setting the test-based policy should probably remain restricted to older children and adults, at least until better and conclusive evidence on its safety in young children is produced. General guidelines on malaria diagnosis and treatment can be misleading, should they fail to take into account the local epidemiology.

## Competing interests

The authors declare that they have no competing interests.

## Authors' contributions

ZB conceived the study design, wrote the study protocol, planned the training of research assistant and wrote the training manual (but for the laboratory). Trained research assistants in rainy season. Supervised enrolment and data collection in the field in rainy season. Concurred to data analysis. Wrote the draft and final version of the manuscript. SBS collaborated to the study design and writing of the study protocol. Contributed to draft versions, revised critically the manuscript.

JM performed statistical analysis (attributable fraction and RDT accuracy on malaria-attributable fever), contributed to draft versions, revised critically the manuscript and approved the final version. CP performed statistical analysis (logistic regression on factors affecting RDT accuracy for malaria infection), contributed to draft versions, revised critically the manuscript. AA trained research assistants in dry season. Supervised enrolment and data collection in the field in dry season. Performed bibliographic research. Revised critically the manuscript. FG trained research assistants in rainy season. Supervised enrolment and data collection in the field in rainy season. Performed bibliographic research. Revised critically the manuscript.

HT contributed to study design. Supervised enrolment and data collection in the field in both seasons. CL carried out first data analyses. Wrote a Master degree thesis (IMTA - Antwerp) reporting part of the paper findings for RDT accuracy for malaria infection. BN enrolled patients, trained and supervised research assistants, coordinated logistics in the field in both seasons. MG trained and supervised research assistants (laboratory) and local laboratory staff; wrote handbook for the research assistants (laboratory); performed microscopy; designed testing of reproducibility of microscopy reading. JVdE contributed with major inputs to the study design. Critically reviewed all draft versions, extensively collaborating for the final version.

All authors read and approved the final version of the manuscript.

## Supplementary Material

Additional file 1 **Supplement Table 1**. Calculation of diagnostic accuracy of RDT for malaria - attributable fever during the low transmission season Click here for file

Additional file 2 **Supplement Table 2**. Calculation of diagnostic accuracy of RDT for malaria - attributable fever during the high transmission season Click here for file

Additional file 3 **Supplement Table 3**. Diagnostic accuracy of RDT for malaria - attributable fever during the low transmission season based on individual level logistic regression models Click here for file

Additional file 4 **Supplement Table 4**. Diagnostic accuracy of RDT for malaria - attributable fever during the high transmission season based on individual level logistic regression models Click here for file
