# Supplementary material for: Accuracy of a rapid diagnostic test on the diagnosis of malaria infection and of malaria - attributable fever during low and high transmission season in Burkina Faso
Source: Malar J. 2010 Jul 7;9:192. doi: 10.1186/1475-2875-9-192 (PMC2914059; doi:10.1186/1475-2875-9-192)
Supplement: Additional file 1 — Supplement Table 1. Calculation of diagnostic accuracy of RDT for malaria - attributable fever during the low transmission season [file 1475-2875-9-192-S1.DOC]

**Supplement Table 1: Calculation of diagnostic accuracy of RDT for clinical malaria in febrile patients during the low transmission season**

| Age | Parasite density | Febrile |  | Prob RDT | Clinical malaria | | Not clinical malaria | | SE | SP | PPV | NPV |
| --- | --- | --- | --- | --- | --- | --- | --- | --- | --- | --- | --- | --- |
| (years) | (/µL) | N | AF | + | TP  (a) | FN  (b) | FP  (c) | TN  (d) | $\frac{a}{a+b}$ | $\frac{d}{c+d}$ | $\frac{a}{a+c}$ | $\frac{d}{b+d}$ |
| <1 | 0 | 110 | 0 | 0.10 | 0 | 0 | 11.5 | 98.5 |  |  |  |  |
|  | 1-400 | 16 | 0.06 | 0.76 | 0.7 | 0.2 | 11.5 | 3.6 |  |  |  |  |
|  | 401-4000 | 9 | 0 | 0.96 | 0 | 0 | 8.7 | 0.3 |  |  |  |  |
|  | 4001-40000 | 5 | 0.24 | 1.00 | 1.2 | 0 | 3.8 | 0 |  |  |  |  |
|  | 40000+ | 3 | 0.86 | 1.00 | 2.5 | 0 | 0.4 | 0 |  |  |  |  |
| 1 - 4 | 0 | 214 | 0 | 0.10 | 0 | 0 | 22.4 | 191.6 |  |  |  |  |
|  | 1-400 | 39 | 0.06 | 0.76 | 1.7 | 0.5 | 28.0 | 8.8 |  |  |  |  |
|  | 401-4000 | 29 | 0 | 0.96 | 0 | 0 | 28.0 | 2.0 |  |  |  |  |
|  | 4001-40000 | 12 | 0.24 | 1.00 | 2.8 | 0 | 9.2 | 0 |  |  |  |  |
|  | 40000+ | 5 | 0.86 | 1.00 | 4.3 | 0 | 0.7 | 0 |  |  |  |  |
| 5 - 14 | 0 | 90 | 0 | 0.10 | 0 | 0 | 9.4 | 80.6 |  |  |  |  |
|  | 1-400 | 21 | 0.06 | 0.76 | 0.9 | 0.3 | 15.1 | 4.8 |  |  |  |  |
|  | 401-4000 | 11 | 0 | 0.96 | 0 | 0 | 10.6 | 0.4 |  |  |  |  |
|  | 4001-40000 | 6 | 0.24 | 1.00 | 1.4 | 0 | 4.6 | 0 |  |  |  |  |
|  | 40000+ | 2 | 0.86 | 1.00 | 1.7 | 0 | 0.3 | 0 |  |  |  |  |
| 15+ | 0 | 252 | 0 | 0.10 | 0 | 0 | 26.3 | 225.6 |  |  |  |  |
|  | 1-400 | 23 | 0.06 | 0.76 | 1.0 | 0.3 | 16.5 | 5.2 |  |  |  |  |
|  | 401-4000 | 1 | 0 | 0.96 | 0 | 0 | 1.0 | 0 |  |  |  |  |
|  | 4001-40000 | 3 | 0.24 | 1.00 | 0.7 | 0 | 2.3 | 0 |  |  |  |  |
|  | 40000+ | 1 | 0.86 | 1.00 | 0.9 | 0 | 0.1 | 0 |  |  |  |  |
| <1 | | 143 |  |  | 4.4 | 0.2 | 35.9 | 102.4 | 95 | 74 | 11 | 99.8 |
| 1 - 4 | | 299 |  |  | 8.8 | 0.5 | 88.2 | 201.5 | 94 | 70 | 9 | 99.7 |
| 5 - 14 | | 130 |  |  | 4.0 | 0.3 | 39.9 | 85.7 | 93 | 68 | 9 | 99.7 |
| 15+ | | 280 |  |  | 2.5 | 0.3 | 46.2 | 230.9 | 89 | 83 | 5 | 99.9 |
| All | | 852 |  |  | 19.8 | 1.3 | 210.3 | 620.6 | 94 | 75 | 9 | 99.8 |

N: number of febrile patients in each age-parasite density combination. AF: Attributable fraction of fever cases to malaria. Prob RDT +: Probability for an RDT positive result.
TP, FN, FP, TN: expected number of true positives, false negatives, false positives and true negatives of the RDT for clinical malaria diagnosis among the N febrile cases in each age-parasite density combination. Estimates obtained from N, AF and Prob RDT + (see methods).Numbers presented are rounded to 1 decimal place; actual calculations based on a better numerical precision.

SE, SP, PPV, NPV: estimated sensitivity, specificity, positive predictive value, negative predictive value of RDT for clinical malaria.
