# Supplementary material for: Accuracy of a rapid diagnostic test on the diagnosis of malaria infection and of malaria - attributable fever during low and high transmission season in Burkina Faso
Source: Malar J. 2010 Jul 7;9:192. doi: 10.1186/1475-2875-9-192 (PMC2914059; doi:10.1186/1475-2875-9-192)
Supplement: Additional file 2 — Supplement Table 2. Calculation of diagnostic accuracy of RDT for malaria - attributable fever during the high transmission season [file 1475-2875-9-192-S2.DOC]

**Supplement Table 2: Calculation of diagnostic accuracy of RDT for for malaria – attributable fever during the high transmission season**

| Age | Parasite density | Febrile |  | Prob RDT | Clinical malaria | | Not clinical malaria | | SE | SP | PPV | NPV |
| --- | --- | --- | --- | --- | --- | --- | --- | --- | --- | --- | --- | --- |
| (years) | (/µL) | N | AF | + | TP  (a) | FN  (b) | FP  (c) | TN  (d) | $\frac{a}{a+b}$ | $\frac{d}{c+d}$ | $\frac{a}{a+c}$ | $\frac{d}{b+d}$ |
| <1 | 0 | 27 | 0 | 0.40 | 0 | 0 | 10.8 | 16.2 |  |  |  |  |
|  | 1-400 | 14 | 0.94 | 0.89 | 11.7 | 1.5 | 0.8 | 0.1 |  |  |  |  |
|  | 401-4000 | 40 | 0.81 | 0.94 | 30.6 | 1.8 | 7.2 | 0.4 |  |  |  |  |
|  | 4001-40000 | 41 | 0.92 | 1.00 | 37.6 | 0 | 3.4 | 0 |  |  |  |  |
|  | 40000+ | 37 | 0.91 | 1.00 | 33.6 | 0 | 3.4 | 0 |  |  |  |  |
| 1 - 4 | 0 | 111 | 0 | 0.39 | 0 | 0 | 42.8 | 68.2 |  |  |  |  |
|  | 1-400 | 40 | 0.56 | 0.83 | 18.6 | 3.7 | 14.7 | 2.9 |  |  |  |  |
|  | 401-4000 | 132 | 0.69 | 0.96 | 87.7 | 4.0 | 38.6 | 1.8 |  |  |  |  |
|  | 4001-40000 | 100 | 0.74 | 1.00 | 73.5 | 0 | 26.5 | 0 |  |  |  |  |
|  | 40000+ | 121 | 0.94 | 1.00 | 113.4 | 0 | 7.6 | 0 |  |  |  |  |
| 5 - 14 | 0 | 77 | 0 | 0.36 | 0 | 0 | 27.1 | 49.9 |  |  |  |  |
|  | 1-400 | 23 | 0 | 0.82 | 0 | 0 | 28.0 | 6.0 |  |  |  |  |
|  | 401-4000 | 37 | 0.47 | 0.93 | 24.9 | 1.8 | 28.3 | 2.0 |  |  |  |  |
|  | 4001-40000 | 21 | 0.73 | 1.00 | 38.0 | 0 | 14.0 | 0 |  |  |  |  |
|  | 40000+ | 25 | 0.96 | 0.96 | 50.5 | 2.2 | 2.2 | 0.1 |  |  |  |  |
| 15+ | 0 | 261 | 0 | 0.08 | 0 | 0 | 20.9 | 240.1 |  |  |  |  |
|  | 1-400 | 36 | 0 | 0.58 | 0 | 0 | 20.8 | 15.2 |  |  |  |  |
|  | 401-4000 | 36 | 0.40 | 0.90 | 12.9 | 1.4 | 19.5 | 2.2 |  |  |  |  |
|  | 4001-40000 | 41 | 0.69 | 1.00 | 28.4 | 0 | 12.6 | 0 |  |  |  |  |
|  | 40000+ | 5 | 0.88 | 1.00 | 4.4 | 0 | 0.6 | 0 |  |  |  |  |
| <1 | | 159 |  |  | 113.6 | 3.3 | 25.5 | 16.7 | 97 | 40 | 82 | 84 |
| 1 - 4 | | 504 |  |  | 293.2 | 7.7 | 130.2 | 72.9 | 97 | 36 | 69 | 90 |
| 5 - 14 | | 275 |  |  | 113.4 | 4.0 | 99.6 | 58.1 | 97 | 37 | 53 | 94 |
| 15+ | | 379 |  |  | 45.7 | 1.4 | 74.4 | 257.4 | 97 | 78 | 38 | 99.4 |
| All | | 1317 |  |  | 565.8 | 16.4 | 329.7 | 405.1 | 97 | 55 | 63 | 96 |

N: number of febrile patients in each age-parasite density combination. AF: Attributable fraction of fever cases to malaria. Prob RDT +: Probability for an RDT positive result.
TP, FN, FP, TN: expected number of true positives, false negatives, false positives and true negatives of the RDT for clinical malaria diagnosis among the N febrile cases in each age-parasite density combination. Estimates obtained from N, AF and Prob RDT + (see methods).Numbers presented are rounded to 1 decimal place; actual calculations based on a better numerical precision.

SE, SP, PPV, NPV: estimated sensitivity, specificity, positive predictive value, negative predictive value of RDT for clinical malaria.
