# Supplementary material for: Accuracy of a rapid diagnostic test on the diagnosis of malaria infection and of malaria - attributable fever during low and high transmission season in Burkina Faso
Source: Malar J. 2010 Jul 7;9:192. doi: 10.1186/1475-2875-9-192 (PMC2914059; doi:10.1186/1475-2875-9-192)
Supplement: Additional file 3 — Supplement Table 3. Diagnostic accuracy of RDT for malaria - attributable fever during the low transmission season based on individual level logistic regression models [file 1475-2875-9-192-S3.DOC]

**Supplement Table 3: Diagnostic accuracy of RDT for malaria – attributable fever during the low transmission season based on individual level logistic regression models**

| Age | Parasite density | Febrile | Clinical malaria | | Not clinical malaria | | SE | SP | PPV | NPV |
| --- | --- | --- | --- | --- | --- | --- | --- | --- | --- | --- |
| (years) | (/µL) | N | TP  (a) | FN  (b) | FP  (c) | TN  (d) | $\frac{a}{a+b}$ | $\frac{d}{c+d}$ | $\frac{a}{a+c}$ | $\frac{d}{b+d}$ |
| <1 | | 143 | 2.0 | 0.0 | 44.5 | 96.5 | 99 | 68 | 4 | 100 |
| 1 - 4 | | 299 | 4.2 | 0.0 | 104.4 | 190.3 | 99 | 65 | 4 | 100 |
| 5 - 14 | | 130 | 1.5 | 0.0 | 45.3 | 83.2 | 99 | 65 | 3 | 100 |
| 15+ | | 280 | 0.7 | 0.1 | 30.0 | 249.3 | 92 | 89 | 2 | 100 |
| All | | 852 | 8.3 | 0.1 | 224.2 | 619.4 | 99 | 73 | 4 | 100 |

N: number of febrile patients in each age-parasite density combination. AF: Attributable fraction of fever cases to malaria. Prob RDT +: Probability for an RDT positive result.
TP, FN, FP, TN: expected number of true positives, false negatives, false positives and true negatives of the RDT for clinical malaria diagnosis among the N febrile cases in each age-parasite density combination. Estimates obtained from N, AF and Prob RDT + (see methods).Numbers presented are rounded to 1 decimal place; actual calculations based on a better numerical precision.

SE, SP, PPV, NPV: estimated sensitivity, specificity, positive predictive value, negative predictive value of RDT for clinical malaria.
