# Supplementary material for: Accuracy of a rapid diagnostic test on the diagnosis of malaria infection and of malaria - attributable fever during low and high transmission season in Burkina Faso
Source: Malar J. 2010 Jul 7;9:192. doi: 10.1186/1475-2875-9-192 (PMC2914059; doi:10.1186/1475-2875-9-192)
Supplement: Additional file 4 — Supplement Table 4. Diagnostic accuracy of RDT for malaria - attributable fever during the high transmission season based on individual level logistic regression models [file 1475-2875-9-192-S4.DOC]

**Supplement Table 4: Diagnostic accuracy of RDT for malaria – attributable fever during the high transmission season based on individual level logistic regression models**

| Age | Parasite density | Febrile | Clinical malaria | | Not clinical malaria | | SE | SP | PPV | NPV |
| --- | --- | --- | --- | --- | --- | --- | --- | --- | --- | --- |
| (years) | (/µL) | N | TP  (a) | FN  (b) | FP  (c) | TN  (d) | $\frac{a}{a+b}$ | $\frac{d}{c+d}$ | $\frac{a}{a+c}$ | $\frac{d}{b+d}$ |
| <1 | | 159 | 101.8 | 2.9 | 37.5 | 16.7 | 97 | 31 | 73 | 85 |
| 1 - 4 | | 504 | 296.4 | 8.6 | 126.5 | 72.5 | 97 | 36 | 70 | 89 |
| 5 - 14 | | 275 | 125.7 | 3.0 | 82.2 | 64.1 | 98 | 44 | 60 | 95 |
| 15+ | | 379 | 42.8 | 2.1 | 82.5 | 251.6 | 95 | 75 | 34 | 99 |
| All | | 1317 | 566.7 | 16.6 | 328.7 | 405.0 | 97 | 55 | 63 | 96 |

N: number of febrile patients in each age-parasite density combination. AF: Attributable fraction of fever cases to malaria. Prob RDT +: Probability for an RDT positive result.
TP, FN, FP, TN: expected number of true positives, false negatives, false positives and true negatives of the RDT for clinical malaria diagnosis among the N febrile cases in each age-parasite density combination. Estimates obtained from N, AF and Prob RDT + (see methods).Numbers presented are rounded to 1 decimal place; actual calculations based on a better numerical precision.

SE, SP, PPV, NPV: estimated sensitivity, specificity, positive predictive value, negative predictive value of RDT for clinical malaria.
